# Anchor free based Siamese network tracker with transformer for RGB-T tracking

**DOI:** 10.1038/s41598-023-39978-7

**Published:** 2023-08-16

**Authors:** Liangsong Fan, Pyeoungkee Kim

**Affiliations:** 1 Silla University, 140, Baekyang-daero 700beon-gil, Sasang-gu, 46958, Busan, Korea; 2 Jilin Institute of Chemical Technology, No. 45 Chengde Street, Jilin City, 132022 Jilin Province China

**Keywords:** Computer science, Software, Optics and photonics

## Abstract

In recent years, many RGB-THERMAL tracking methods have been proposed to meet the needs of single object tracking under different conditions. However, these trackers are based on ANCHOR-BASED algorithms and feature cross-correlation operations, making it difficult to improve the success rate of target tracking. We propose a siamAFTS tracking network, which is based on ANCHOR-FREE and utilizes a fully convolutional training network with a Transformer module, suitable for RGB-THERMAL target tracking. This model addresses the issue of low success rate in current mainstream algorithms. We also incorporate channel and channel spatial attention modules into the network to reduce background interference on predicted bounding boxes. Unlike current ANCHOR-BASED trackers such as MANET, DAPNet, SGT, and ADNet, the proposed framework eliminates the use of anchor points, avoiding the challenges of anchor hyperparameter tuning and reducing human intervention. Through repeated experiments on three datasets, we ultimately demonstrate the improved success rate of target tracking achieved by our proposed tracking network.

## Introduction

In order to predict and track the next location of a target, target tracking algorithms typically start with a specific initial target position feature, establish it as a reference, and then perform correlation operations with consecutive frames. Most tracking trackers are based on RGB images. However, in some challenging conditions such as fog, rain, and darkness, where the target is not clearly visible in RGB images, the tracker often fails to successfully track the target. In recent years, there has been increasing research interest in multi-modal trackers that combine other modalities with RGB images, such as RGB-T tracking, to address this limitation. To enhance tracking performance, Li et al. proposed a method based on the fusion of grayscale and thermal image categories^1^. Furthermore, they publicly shared their dataset in the paper. RGB-THERMAL trackers are more competitive as they leverage the complementary advantages of the fused RGB and thermal modes. In this approach, thermal infrared images are unaffected by lighting conditions, while RGB photographs capture detailed information pertaining to the target.

Since Li^2^ successfully improved the accuracy of target tracking by using Siamese networks, which extract features from two identical branching networks, this Siamese network has been widely applied in the design of RGB-THERMAL target tracking^6–9^ and^10^. all demonstrate that Siamese networks can effectively improve the accuracy and success rate of target tracking. In this study, we recommend the twin network for RGB-THERMAL fusion tracking, primarily for the tracking network's stability consideration.

In recent years, several different RGB-THERMAL tracking techniques have been introduced. Xingchen Zhang et al.^3^ employed a fully convolutional network and multilayer feature fusion to enhance thermal tracking performance. Guo et al.^4^ utilized a deep network model and combined RGB and thermal heat score maps to increase tracking speed. Zhu^5^ proposed a novel concept that emphasizes the importance of layers capturing key features and combined the separately collected layers to produce better prediction results.

The mentioned RGB-T trackers and many advanced RGB-T models^11–14^ are inspired by the Siam RPN^8^ model, which primarily involves predefining anchor frames of different sizes^6,9^ that each sample must match, leading to increased computation and processing time. These anchor frames are artificially designed and have a significant impact on the effectiveness of the tracking model, requiring a considerable amount of manual intervention during the experimental process. How to enable network models to learn autonomously without human intervention is also a current direction of development in artificial intelligence.

Currently, Siamese networks are widely used in advanced RGBT tracking algorithms. Siamese networks typically perform correlation operations on image features extracted from the target template and search region, and determine their similarity based on the similarity score. The target object is considered successfully tracked if the similarity score is high. However, correlation operations, which involve convolving two feature maps, may result in significant information loss, leading to a low success rate in target tracking. Recently, in the field of RGB-based target tracking, many researchers have incorporated Transformer technology into the design of tracking algorithms and achieved promising results. However, there are few studies that apply Transformer technology to RGBT-based target tracking, and the accuracy and success rate of RGBT trackers using Transformer are not high. Inspired by the application of Transformer in target tracking, we aim to design a new model with a Transformer framework to improve the accuracy and success rate of RGBT target tracking.

This study proposes an end-to-end trainable tracker based on Transformer for robust RGBT tracking. Firstly, we extract features from RGB and thermal infrared images separately. Then, we employ spatial and channel attention modules to enhance these features and improve the resolution of fused features, effectively reducing the discrepancies between modality features and eliminating background interference. Finally, by fusing these image features, we obtain a response map through our newly designed Transformer module. Our novel model predicts the target's position and bounding box using only one response map. In summary, our main contributions include:

1. We designed an end-to-end offline tracking training model by using convolutional neural networks. Previous RGBT designs have been anchor-based; in this case, our model is anchor-free, which does not depend on pre-defined boxes and can be trained on well-annotated datasets and achieve good results.
2. We have designed a new Transformer module specifically for RGBT target tracking. Experimental results demonstrate that the use of this module significantly improves the accuracy and success rate of target tracking. Our designed tracker takes into account the contributions of both RGB and TIR modes in modeling the target. It effectively utilizes the feature information to enhance the robustness of the model.
3. We conducted an in-depth analysis of the GTOT, GBT210, and RGBT234 datasets. The results show that our proposed method exhibits some gaps when compared to the latest supervised RGBT algorithms. However, our tracker demonstrates highly competitive performance in many aspects.

## Proposed method

**Figure 1:**
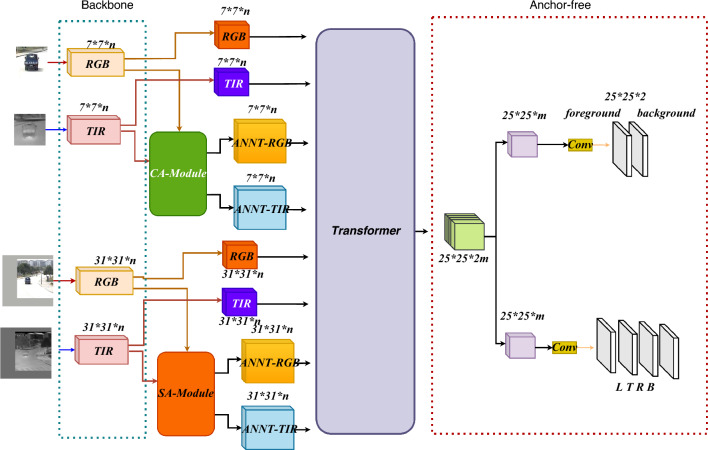
The frame construction.

Figure 1 depicts the overall frame construction. We outline each part of our technique separately in this section.

### Backbone

As shown in Fig. 1 as the input of 4 images, our input consists of a visible light branch and a thermal infrared branch. The target branch in turn contains visible input Z1 and thermal infrared input Z2, and the search area branch is divided into visible input X1 and thermal infrared input X2. Since it is a Siamese model, both the visible and thermal infrared branches use the same ResNet50 model to extract feature maps. After backbone network feature extraction, the visible branch will get feature maps $\varphi (Z1)$ and feature maps $\varphi (X1)$,and the thermal infrared branch will get feature maps $\varphi (Z2)$ and feature maps $\varphi (X2)$. Then, we obtain the feature maps $\varphi (Z1)$,$\varphi (X1)$,$\varphi (Z2)$,and $\varphi (X2)$, where a portion is directly fed as input to the Transformer module for the next step of computation, while another portion is enhanced by passing through the SA and CA modules to augment the feature maps before being transmitted to the Transformer module for further computation.

During the object tracking process, we aim to include more image feature information in the response map. Inspired by reference^7^, we consider extracting feature maps from different layers as outputs during the process of feature extraction. Deep features and shallow features have different roles in target tracking. First, deep features have good discrimination of the required speech properties, which are enhanced for our classification task. In contrast, shallow features are rich in information about visual attributes such as edges and colors, which are enhanced for the target localization task. Inspired by references^7,15^, We modify the last module of ResNet50 to obtain feature maps from layers 6, 7, and 8. We obtain F_6_(X1), F_7_(X1), F_8_(X1). And we also get F_6_(X2), F_7_(X2), F_8_(X2). Here 6, 7, and 8 indicates the feature values we extracted from layer 6 layer 7, and layer 8. There are 256 channels in F_6_(X2), F_7_(X2), F_8_(X2).

### Module for spatial attention

**Figure 2:**
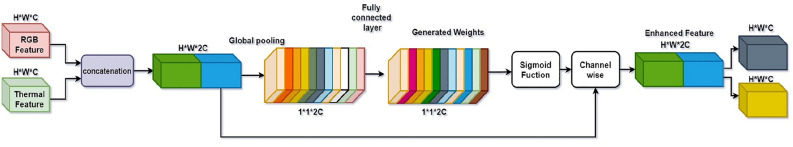
CA model architecture.

The channel attention mechanism can enhance the predictive capability of the network. To improve the information transfer capability between the two modes, we designed a Channel Attention Feature Enhancement module (CA module) as shown in Fig. 2.

In the CA module Fig. 2, we take the feature map $\varphi (Z1)$ and $\varphi (Z2)$ extracted through the backbone network as input to the CA module and obtain the joint feature as $U^{{{\text{ca}}}}$. denoting the output as $x_{rgb}^{ca}$ , $x_{tir}^{ca}$, and the overall CA module can be described as follows:

1 $$x_{rgb}^{ca} ,x_{tir}^{ca} = CA\left( {U^{ca} } \right) = {\text{Split}} {(}U^{ca} \otimes \varepsilon \left( {\omega \left( {\delta \left( {U^{ca} } \right)} \right)} \right)$$

where $\delta$ represents the full set, $\omega$ is the fully connected layer, $\varepsilon$ denotes Sigmoid, $\otimes$ is the product of channel-wise, and Split is the operation of extracting features along the channel dimension.

**Figure 3:**
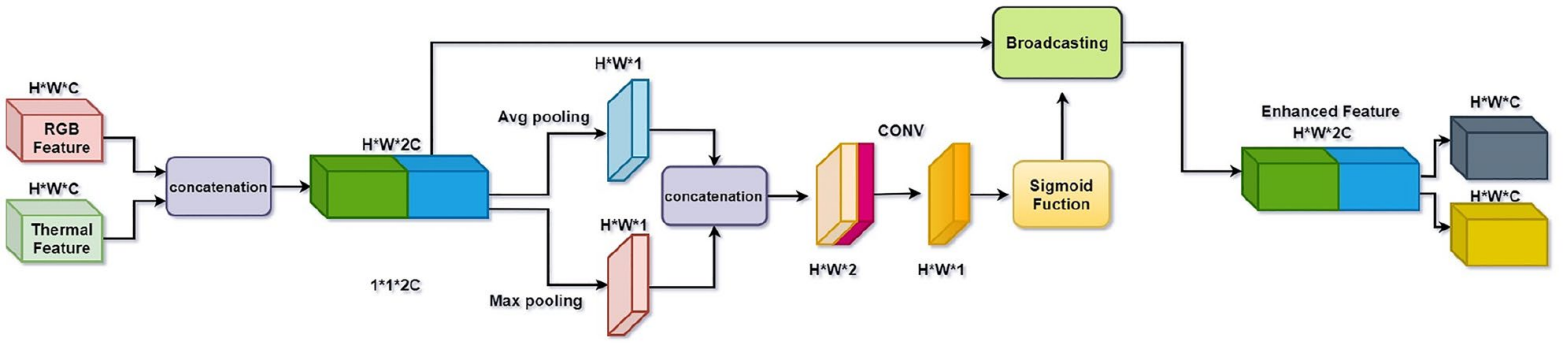
SA model architecture.

In order to suppress the effect of background noise on the classification task, we designed a spatial attention (SA module) module. It is shown in Fig. 3. This module mainly utilizes the spatial inter-relationship of features. We take $\varphi (X1)$ and $\varphi (X2)$ extracted through the backbone network as the input feature maps, and by using the SA module, we finally obtain the feature map $U^{{{\text{ca}}}}$ using the following mathematical expression as:

2 $${\text{ V}}^{sa} = \varepsilon \left( {\varphi \left( {{\text{Cat}} \left( {\rho \left( {U^{sa} } \right),\phi \left( {U^{sa} } \right)} \right),H} \right)} \right)$$

where $\rho$ is for the average set, $\phi$ or the largest set, Cat stands for the process of stringing features along the channel dimension, $\varphi$ stands for the two-dimensional convolution operation, H stands for a collection of kernel weights, and $\varepsilon$ stands for the Sigmoid function.

The output is then represented as $x_{rgb}^{sa} ,x_{tir}^{sa}$, and SA module as seen below:

3 $$x_{rgb}^{sa} ,x_{tir}^{sa} = SA\left( {U^{sa} } \right) = {\text{Split}} \left( {U^{sa} \odot {\text{V}}^{sa} } \right)$$

$\odot$ denotes connecting a CA module to an SA module, and finally, we get the final SA module.

### Transformer network

**Figure 4:**
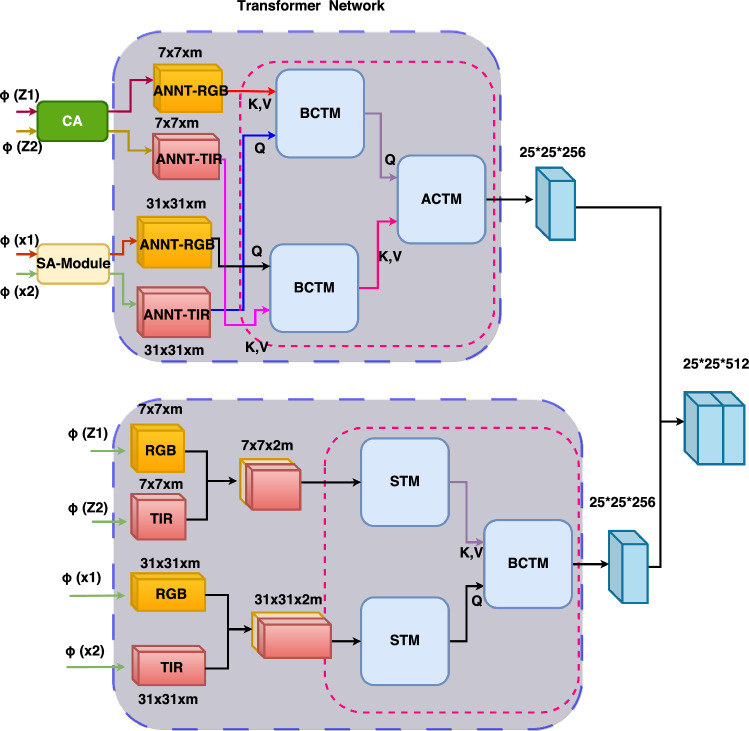
Illustration of the features fusion network based on the transformer.

Inspired by reference^16^, we designed a Transformer Network as shown in Fig. 4.

From Fig. 4, we can see that the template feature and search feature extracted by the backbone network first pass through the CA and SA channel modules respectively, and obtain $x_{rgb}^{sa}$,$x_{tir}^{sa}$,$\widehat{zf}_{rgb}^{ca}$ and $\widehat{zf}_{{{\text{tir}}}}^{ca}$. Then, the feature vectors are fed into the Transformer module, as shown in Fig. 4. First, $x_{rgb}^{sa}$ and $\widehat{zf}_{rgb}^{ca}$ pass through a Transformer attention module (BCTM). Then, $x_{tir}^{sa}$ and $\widehat{zf}_{{{\text{tir}}}}^{ca}$ pass through another Transformer attention module (BCTM). BCTM is used to fuse different branch information. To make the fusion information more accurate, the fusion process is repeated four times. Finally, an extra Transformer module (ACTM) is added to fuse the feature vectors of the template and search branches. BCTM and ACTM have the same network structure. Here, we will provide a detailed explanation using BCTM as an example.

**Figure 5:**
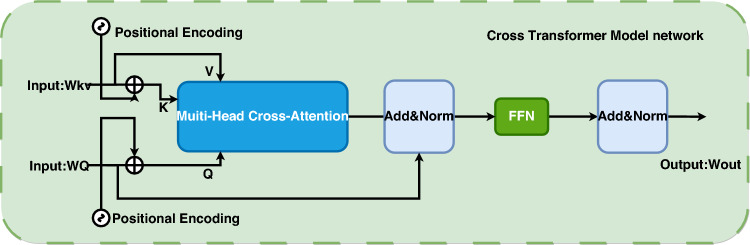
Illustration of the transformer cross transformer module.

Figure 5 shows the BCMT module for transformers. The BCMT module utilizes positional encoding to distinguish position information of feature sequences and utilizes a residual-based multi-head cross-attention to integrate feature vectors from different inputs. Additionally, a residual-based feed-forward network (FFN) is employed to obtain the final output. The specific calculation process of the BCMT module is as follows:

4 $$\begin{gathered} W_{CF}{\prime} = W_{Q} + MultiHead(W_{Q} + P_{Q} ,W_{KV} + P_{KV} ,W_{KV} ) \hfill \\ W_{CF} = W_{CF}{\prime} + FFN(W_{CF}{\prime} ) \hfill \\ \end{gathered}$$

The calculation process of the CMT module involves $W \in R^{{{\text{d}} \times N_{x} }}$ and $W_{KV} \in R^{{{\text{d}} \times N_{KV} }}$ as two inputs from different branches, while $P_{Q} \in R^{{{\text{d}} \times N_{Q} }}$ and $P_{KV} \in R^{{{\text{d}} \times N_{KV} }}$ represent spatial positional encodings of $W_{Q}$ and $W_{KV}$. The output of the residual multi-head cross-attention and the final output are represented by $W_{CF}{\prime}$ and $W_{CF}$, respectively.

After ACTM, we obtain enhanced image features R1 of size 25*25*256. Next, referring to the lower part of Fig. 4, we observe that the image features that have not undergone the CA and SA modules are initially fused separately. For example, fusion of features $\varphi (X1)$ and $\varphi (X2)$ results in image feature $\varphi (X)$ of size 31*31*m. Fusion of features $\varphi (Z1)$ and $\varphi (Z2)$ results in image feature $\varphi (Z)$ of size 7*7*m. Firstly, $\varphi (X)$ passes through a Transformer attention module (STM). Then, $\varphi (Z)$ also passes through a Transformer attention module (STM). Finally, we transmit the obtained Q, K, and V to the Transformer attention module (BCTM), from which we can obtain image features R2 of the original image. We perform a CAT operation on R1 and R2, resulting in R:

5 $$R(X) = CAT\left( {R1,R2} \right)$$

**Figure 6:**
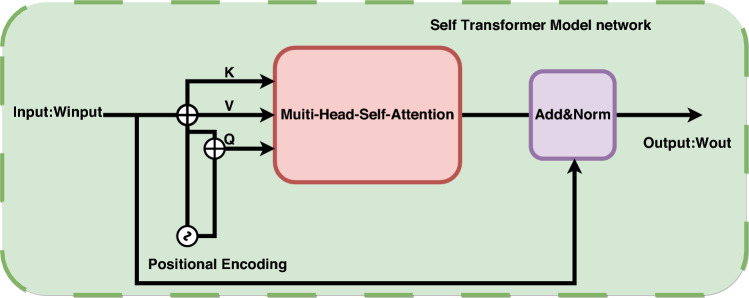
Illustration of the transformer self transformer module.

In Fig. 6, we can observe the self-attention modules for the transformer (STM). These modules begin by incorporating a positional encoding technique to accurately differentiate the position information within feature sequences. Next, they utilize multi-head self-attention to consolidate the feature vectors from various positions. Lastly, a residual form is employed to obtain the output. The specific calculation process of the TS module is described below.

6 $$W_{SF}^{{}} = W_{{}} + MultiHead(W_{{}} + P_{K} ,W_{{}} + P_{K} ,W_{{}} )$$

where $P_{K} \in R^{{{\text{d}} \times N_{x} }}$ denotes the spatial positional encoding obtained through the application of a sine function. $W \in R^{{{\text{d}} \times N_{x} }}$ represents the input to the TS module, while $W_{SF} \in R^{{{\text{d}} \times N_{x} }}$ denotes the resulting output after the TS module's operations.

### Anchor-free based bounding box prediction

#### a. Position prediction head

The location prediction head in Fig. 1 includes classification and regression modules. After passing through the Transformer attention module, we obtain image features of size (*, 256, 25, 25). These features are subsequently used in the location prediction head to generate image features of size (*, 2, 25, 25) and (*, 4, 25, 25) for classification and regression branches, respectively.

#### b. Training loss

Firstly, we classify the input samples into positive and negative samples. Since negative samples have a lower probability of representing the target, we only perform regression operations on positive samples.

We will determine whether a sample is positive or negative by drawing two ellipses, S1 and S2, around the target. We may obtain an ellipse S1 as illustrated in the equation that follows.

7 $$\frac{{\left( {s_{j} - gth_{xc} } \right)^{2} }}{{\left( {\frac{{gth_{w} }}{2}} \right)^{2} }} + \frac{{\left( {s_{j} - gth_{yc} } \right)^{2} }}{{\left( {\frac{{gth_{h} }}{2}} \right)^{2} }} = 1$$

Likewise, we can obtain an ellipse S2:

8 $$\frac{{\left( {s_{j} - gth_{xc} } \right)^{2} }}{{\left( {\frac{{gth_{w} }}{4}} \right)^{2} }} + \frac{{\left( {s_{j} - gth_{yc} } \right)^{2} }}{{\left( {\frac{{gth_{h} }}{4}} \right)^{2} }} = 1$$

If a sample point (k, j) lies outside the ellipse S1, it is defined as a negative sample. Conversely, if it lies inside S2, it is defined as a positive sample.

For the coordinates of positive samples, we perform regression operations. In anchor-based regression, we typically compare predicted boxes with ground truth boxes. However, in the anchor-free regression algorithm we employ, we use the following equation for regression calculations.

9 $$\begin{array}{*{20}l} {d_{l} = s_{j} - gth_{{x_{1} }} } \hfill \\ {d_{t} = s_{k} - gth_{{y_{1} }} } \hfill \\ {d_{r} = gth_{{x_{2} - s_{j} }} } \hfill \\ {d_{b} = gth_{{y_{2} - s_{k} }} } \hfill \\ \end{array}$$

where $d_{l}$, $d_{{\text{t}}}$, $d_{{\text{r}}}$, $d_{{\text{b}}}$ and are the distances from that place to the four edges of the surrounding box. For the calculation of the loss function, we use IOU (Intersection over Union). By modifying the coordinates of the predicted bounding box's top-left corner and bottom-right corner, we can obtain the predicted bounding box for each point on the feature map corresponding to the search image. IOU represents the ratio of the intersection area between the ground truth and the predicted bounding box.

10 $${\text{L}}_{{{\text{IOU}}}} {\text{ = 1 - IOU}}$$

If the regression value exceeds 0 and the point (x, y) marked as a positive sample lies within the ellipse S2, then the IOU value falls between 0 and 1.

### Tracking

The RGBT series consists of visible light and thermal infrared images. The visible light photos and thermal infrared images undergo a cropping process. The size of the search image is adjusted to 255 × 255 pixels, while the size of the template image is adjusted to 127 × 127 pixels. From these images, two sets are selected, each containing 60 samples (40 negative samples and 20 positive samples), which are the visible light and thermal infrared images.

The first step of the prediction process is to set up the tracker, which handles the first frame. Then, we save the image information of the first frame. The search image (second frame) is processed through the backbone to extract feature maps from the 6th, 7th, and 8th layers, which are then resized to 7 × 7. The feature maps extracted from the visible light and thermal infrared images are separately processed using the SA and CA channel attention mechanisms, preparing them for the next step of operations.

We separately input the extracted unenhanced and enhanced visible light and thermal infrared feature maps into the Transformer Network. Then, after undergoing transformation in the Transformer Network, we perform classification and regression operations on the obtained outputs. By performing regression operations using the following equation:

11 $$\begin{array}{*{20}l} {P_{x1} = s_{j} - d_{l}^{reg} } \hfill \\ {P_{y1} = s_{k} - d_{t}^{reg} } \hfill \\ {P_{x2} = d_{r}^{reg} + s_{j} } \hfill \\ {P_{y2} = d_{b}^{reg} + s_{k} } \hfill \\ \end{array}$$

The top and bottom right corners of the prediction box are ($P_{x1}$,$P_{x2}$) and ($P_{y1}$,$P_{y2}$), respectively, while ($d_{l}^{reg}$, $d_{t}^{reg}$,$d_{r}^{reg}$ and $d_{b}^{reg}$) denote the projected values of the regression box. The optimal tracking box is chosen from the generated prediction boxes, and the tracking box coordinates are updated through linear interpolation with the previous frame's state to achieve tracking. After generating the prediction boxes, cosine windows are applied to mitigate significant displacements, and penalties are introduced to discourage substantial changes in size and scale. Through the aforementioned series of operations, we ultimately obtain the best predicted bounding box.

## Experiments

### Data set and device description

In this study, we will evaluate our model by testing it on two datasets, GTOT^1^ , RGBT210^16^ and RGBT234.

Using PyTorch and two GTX 3080-Ti cards for training, the algorithm is put into practice. The search region's input size was 255 pixels, whereas the template's input size was 127 pixels for comparison's sake. we build a training subnetwork with the ResNet-50 as its core. Using ImageNet, the network had already been trained. The pre-trained weights served as an initialization for our model's further training.

### Compared with SOTA RGB-T tackers

#### Compared anchor-based methods

We carefully selected a series of Anchor-Based supervised RGB-T trackers for comparison. These include HMFT^22^, CMPP^23^, DMCNet^27^, JMMAC^26^, CBPNet^33^, MaCNet^25^, MANet^34^, CMP^35^, MFGNet^36^, DAPNet^37^, mfDiMP^38^, LTDA^31^, DuSiamRT^39^, TCNN^29^, JCDA-InvSR^30^, and SiamDW^47^ + RGBT.

To broaden the scope of comparison, we also included transformer-based method APFNet^48^ and non-deep RGB-T trackers, such as CMCF^40^, NRCMR^32^, CMR^41^, SGT^42^, the method proposed by Li et al.^43^, CSR^32,44^, MEET^45^ + RGBT, and KCF^46^ + RGBT. This selection encompasses a wide range of RGB-T methods across various categories.

#### Results on GTOT

The method of this study further concludes the tracking on the GTOT dataset, which contains 50 different video sequences and considers different environmental conditions, as shown in Table 1

**Table 1:** A list of annotated attributes for the GTOT data set.

Attr Description	
OCC	Occlusion
LSV	Large scale variation
FM	Fast motion
LI	Low illumination
LR	Low resolution
TC	Thermal crossover
DEF	Deformation
SO	Small object

**Figure 7:**
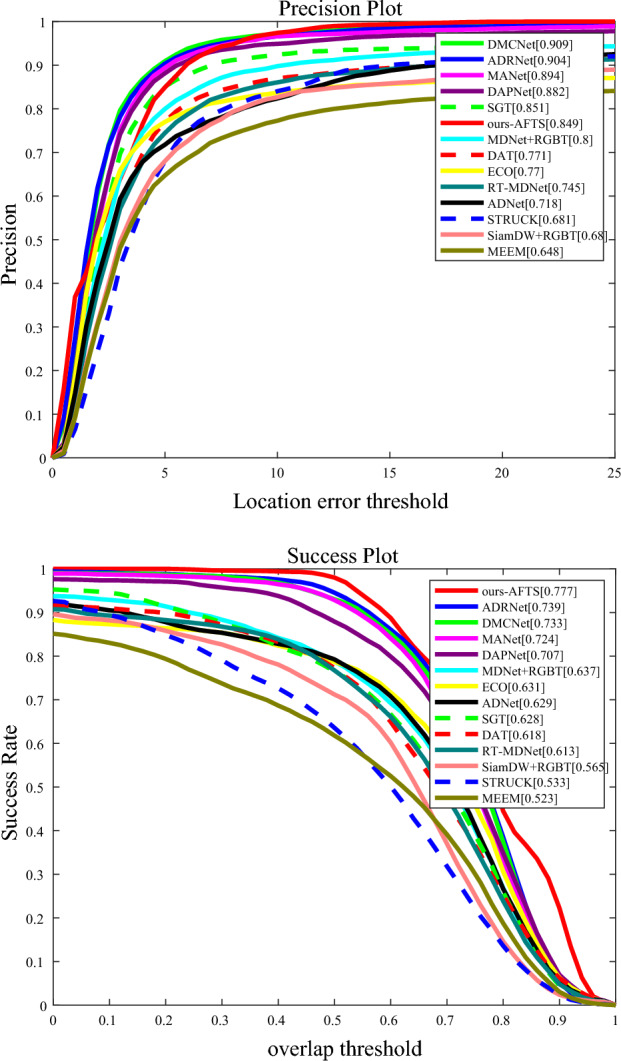
The results of our proposed model compared to other Anchor-Based models on the GTOT datasets, (**a**) Precision Rate; (**b**) Success Rate.

Figure 7 shows the comparison results of our proposed model with other anchor-based models on the GTOT datasets.

Table 3 displays the tracking results on the GTOT dataset. In terms of these two metrics, our RGB-T tracker achieves almost superior success rates compared to all Anchor-Based RGB-T trackers. However, we also notice that our results in accuracy are not high. The performance gap between our supervised RGB-T tracker and the state-of-the-art can be attributed to their usage of large-scale annotated RGB-T image pairs for training. Additionally, these trackers employ more complex models. In the future, we will modify our model to improve accuracy.

#### Results on RGBT210

**Figure 8:**
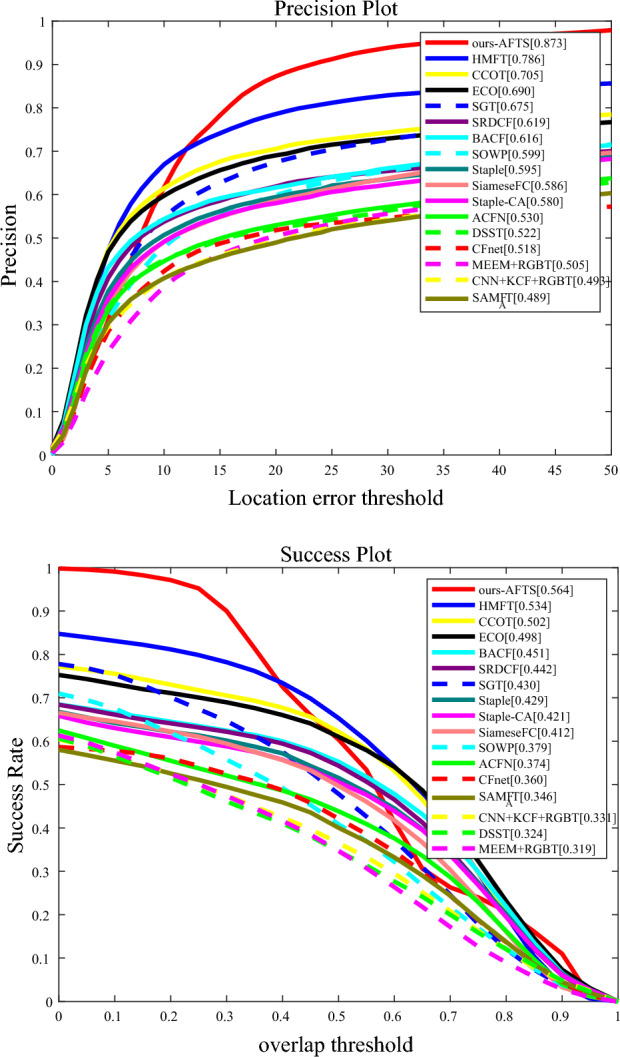
The results of our proposed model compared to other Anchor-Based models on the RGBT210 datasets. (**a**) Precision Rate; (**b**) Success Rate.

The tracking outcomes of this technique using the RGBT210 data set are shown in Fig. 8. 210 real-label visible and thermal infrared video clips are included in RGBT210. This data collection takes a lot of difficult cases into account, as illustrated in Table 2^5^.

**TABLE 2:** List of the attributes annotated to RCBT210.

Attr Description
NO No occlusion—the target is not occluded
PO Partial occlusion—the target object is & partially occluded
HO Heavy occlusion—the target object is & heavy occluded (80% percentage)
LI Low illumination—the illumination in the target region is low
LR Low resolution—the resolution in the target region is low
TC Thermal crossover—the target has a similar temperature to other objects or background surroundings
DEF Deformation—non-rigid object deformation
FM Fast motion—the motion of the ground truth between two adjacent frames are larger than 20 pixels
SV Scale variation—the ratio of the first bounding box and the current bounding box is out of the range [0. 5, 1]
MB Motion blur—the target object's motion results in the blur image information
CM Camera moving—the target object is captured by a moving camera
BC Background clutter—the background information which includes the target object is messy

By performing validation on the RGBT210 dataset, Fig. 8 shows that our tracker beats all trackers.

####  Results on RGBT234

The results on the RGBT234 dataset, presented in Table 3, demonstrate that our proposed RGB-T tracker outperforms both supervised and non-learning-based RGB-T trackers in terms of MSR. However, its performance on RGBT234 is relatively weaker compared to the GTOT dataset. This discrepancy can be attributed to the increased challenges posed by RGBT234, which comprises 234 images and encompasses 12 challenging attributes, surpassing the 7 attributes of GTOT. On the RGBT234 dataset, our model was compared to other Anchor-Based models. The comparison results show that we have achieved almost superior performance compared to all algorithms. However, it is worth noting that we have lower accuracy in certain aspects. To address this performance gap, our future work aims to explore better backbone trackers and larger training datasets.

**Table 3:** Comparison with existing anchor based RGB-T trackers on the GTOT dataset and RGBT234 dataset. The results marked with '∞' are computed by us using raw tracking results. The results marked with '*' are copied from references ^32,48^. Other results are extracted from corresponding papers. '––' means not mentioned in the corresponding paper. Values worse than our method are marked in pink and yellow.

Tracker	GTOT		RGBT234		Category	Supervised	Venue
	MPR(↑)	MSR(↑)	MPR(↑)	MSR(↑)			
HMFT∞^22^	90.6	74.2	78.8	56.8	DL-based	Yes	CVPR 2022
CMPP^23^	92.6	73.8	82.3	57.5	DL-based	Yes	CVPR 2020
APFNet^24^	90.5	73.7	82.7	57.9	DL-based	Yes	AAAI 2022
DMCNet ∞^27^	90.9	73.3	83.9	59.3	DL-based	Yes	IEEE TNNLS 2022
JMMAC^26^	89.3	73.1	79.0	57.3	DL-based	Yes	IEEE TIP 2021
CBPNet^33^	88.5	71.6	79.4	54.1	DL-based	Yes	IEEE TMM 2021
MaCNet^25^	88.0	71.4	79.0	55.4	DL-based	Yes	Sensors 2020
MANet ∞^34^	88.9	71.1	77.7	53.9	DL-based	Yes	ICCVW 2019
CMP^35^	86.9	71.1	75.1	49.1	DL-based	Yes	Neurocomputing 2021
MFGNet^36^	88.9	70.7	78.3	53.5	DL-based	Yes	IEEE TMM 2022
DAPNet∞^37^	87.4	68.9	76.6	53.7	DL-based	Yes	ACM MM 2019
mfDiMPS*^38^	84.1	69.3	78.5	55.9	DL-based	Yes	ICCVW 2019
LTDA^31^	84.3	67.7	78.7	54.5	DL-based	Yes	ICIP 2019
DuSiamRT^39^	76.6	62.8	56.7	38.4	DL-based	Yes	The Visual Computer 2022
TCNN^29^	85.2	62.6	–	–	DL-based	Yes	Neurocomputing 2018
JCDA-InvSR^30^	–	60.5	60.6	41.4	ML-based	Yes	IEEE TIP 2019
SiamDw^47^ + RGBTS*	68.0	56.5	60.4	39.7	DL-based	Yes	CVPR 2019
CMCF^40^	77.0	63.2	–	–	Non-DL (CF-based)		Neurocomputing 2019
NRCMR^32^	83.7	66.4	72.9	50.2	Non-DL (Graph-based)		IEEE TNNLS 2021
CMR^41^	82.7	64.3	–	–	Non-DL (Graph-based)		ECCV 2018
SGTS ∞^42^	85.1	62.8	72.0	47.2	Non-DL (Graph-based)		ACM MM 2017
^43^	84.2	62.2	–	–	Non-DL (Graph-based)		SPIC 2018
CSR ∞^28^	74.5	61.5	46.3	32.8	Non-DL (SR-based)		IEEE TIP 2016
^44^	77.3	61.2	72.9	48.6	Non-DL(Graph-based)		Neurocomputing 2022
MEEM^45^ + RGBTS∞	–	52.0	63.6	40.5	Non-DL		ECCV 2014
KCF^46^ + RGBTS*	–	42.0	46.3	30.5	Non-DL (CF-based)		IEEE TPAMI 2014
OURS	84.9	77.7	89.0	60.2	DL-based	Yes	

**Figure 9:**
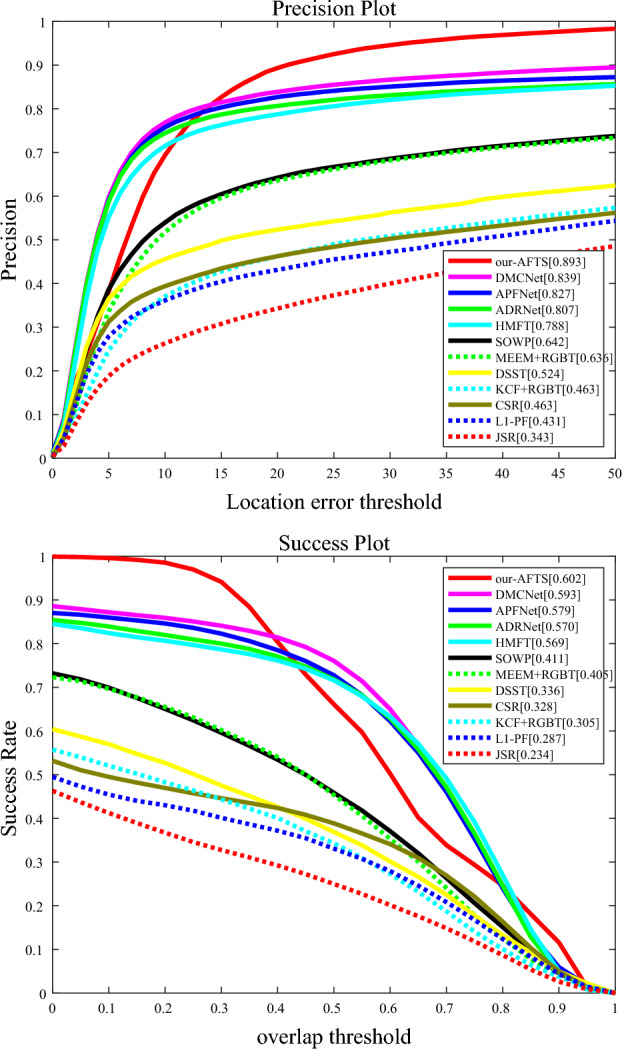
The results of our proposed model compared to other Anchor-Based models on the RGBT234 datasets. (**a**) Precision Rate; (**b**) Success Rate.

By performing validation on the RGBT234 dataset, Fig. 9 shows that our tracker beats all trackers.

#### Attribute-based results

**Figure 10:**
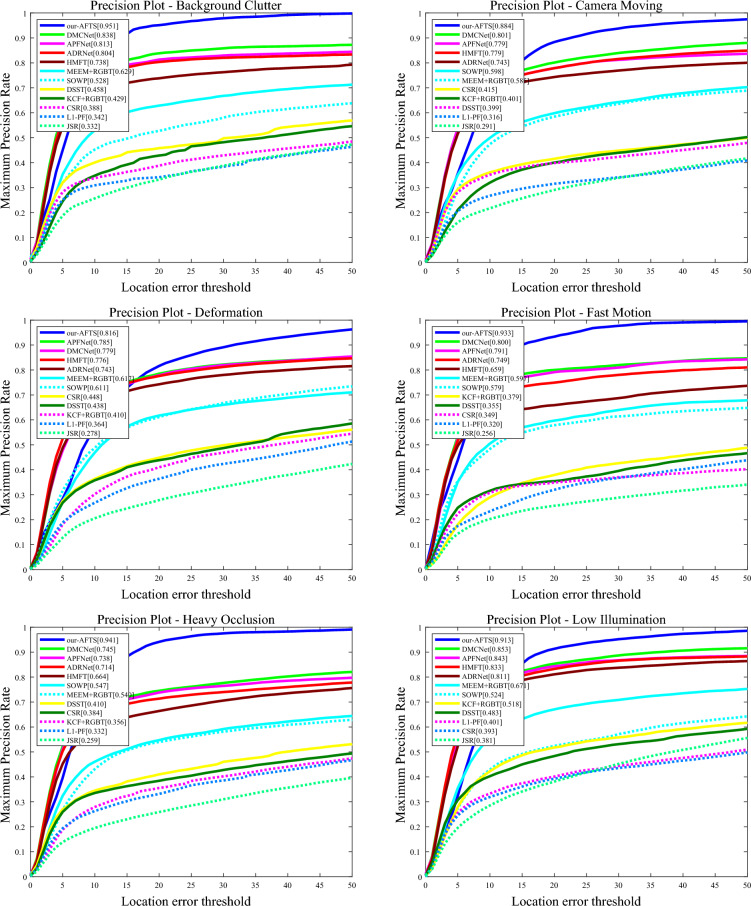

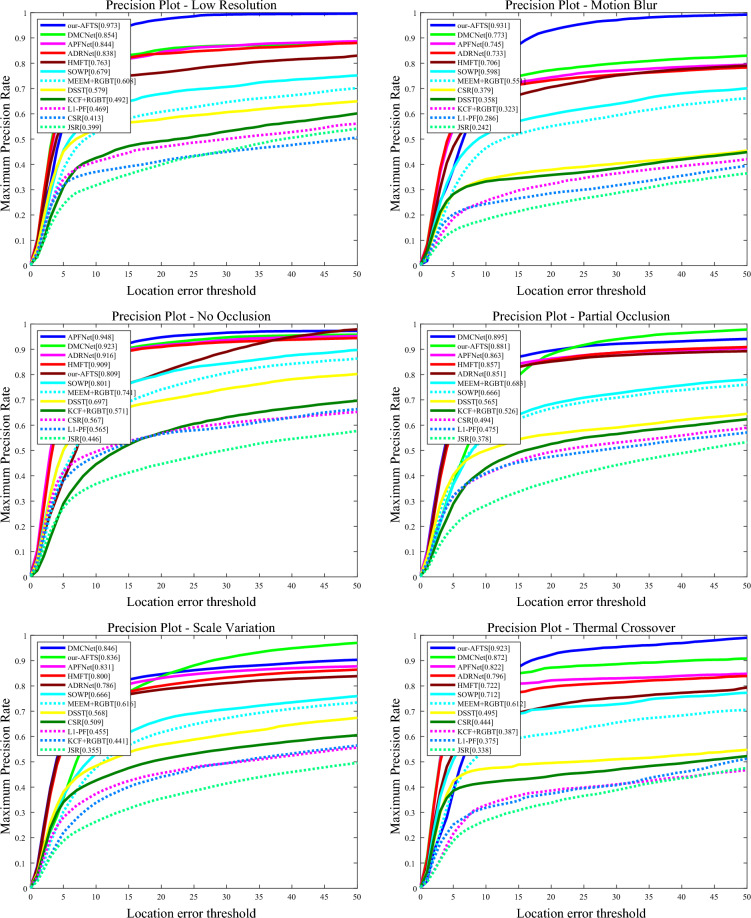
The figure illustrates the comparison of our model's accuracy against other state-of-the-art Anchor-Based models on 12 challenging attributes in the RGBT234 dataset.

**Figure 11:**
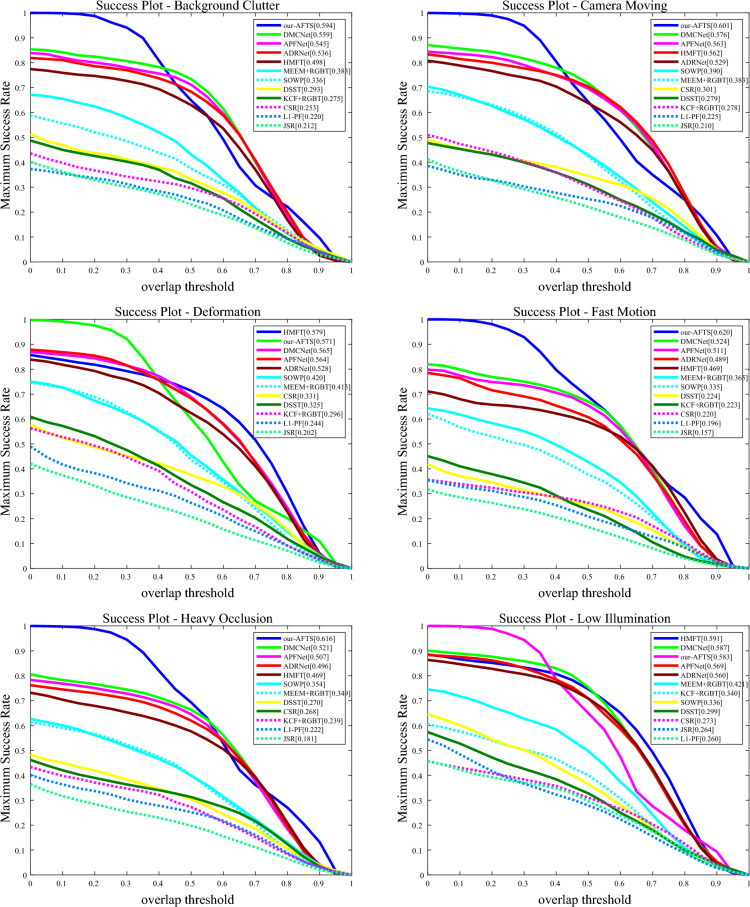

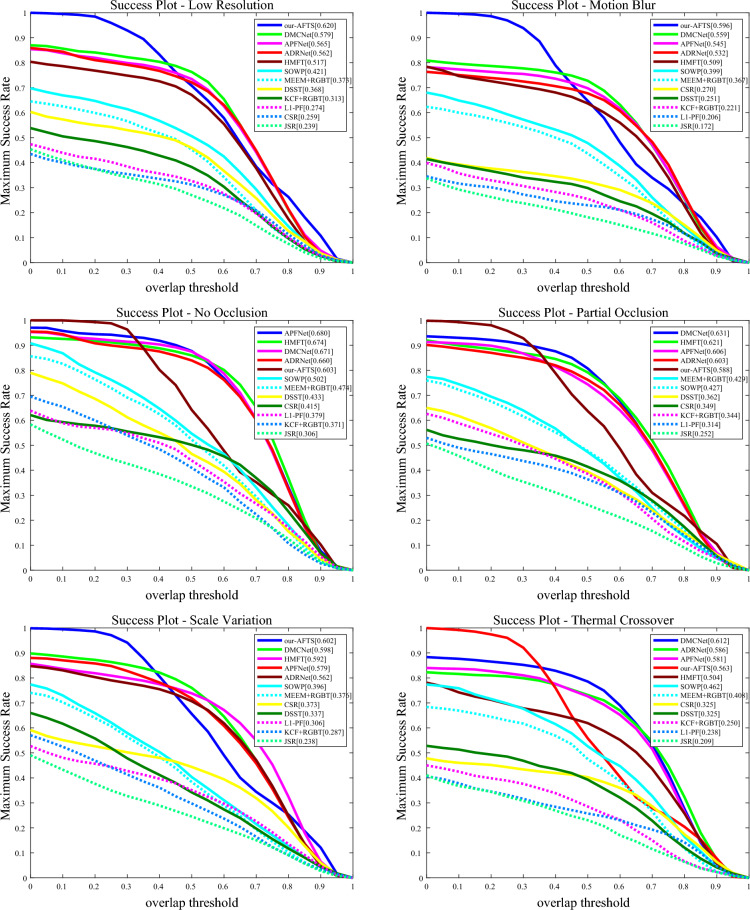
The figure demonstrates the comparison of our model's success rate against other state-of-the-art Anchor-Based models on 12 challenging attributes in the RGBT234 dataset.

The performance of the challenging attributes on the RGBT234 dataset is shown in Figs. 10 and 11.

It can be observed that our RGB-T tracker achieves highly competitive performance in various aspects. In the MSR graph of the challenging attributes, our model performs well in most attributes except for TC, PO, NO, and LI, where it is not as effective as other models. However, our model performs well in the remaining attributes. In the MPR graph of the challenging attributes, our model performs poorly in SV, PO, and NO, but demonstrates excellent performance in the other nine attributes. Overall, our model faces challenges in dealing with PO and NO, indicating areas for improvement in our future work.

#### Qualitative results

**Figure 12:**
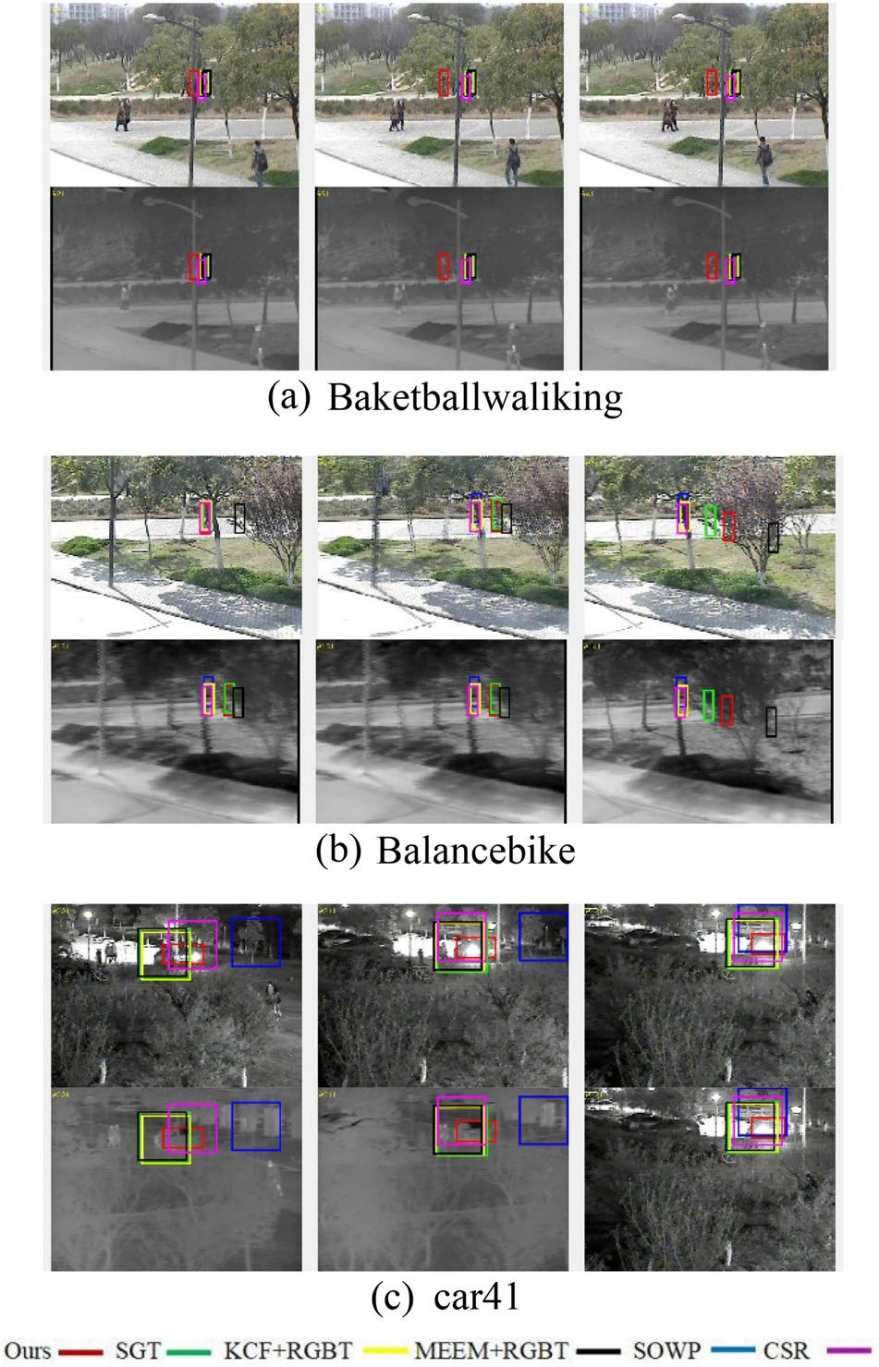
Results of an experimental assessment of the RGBT210.

As shown in Fig. 12, our RGB-T tracker is compared qualitatively with other anchor-based RGB-T trackers on the RGBT210 dataset. The images in Fig. 12 are sourced from the RGBT210 dataset^17^. We would like to express our gratitude to LI^17^ for making the dataset publicly available. We selected several RGB-T trackers, including SOWP^18^, SOWP + RGBT, KCF^19^ + RGBT, CSR^20^, SGT, and MEEM^21^ + RGBT. It can be seen from the figure that our RGB-T tracker performs better on these three sequences (Baketballwaliking , Balancebike, car41).

###  Ablation studies and analysis

**Figure 13:**
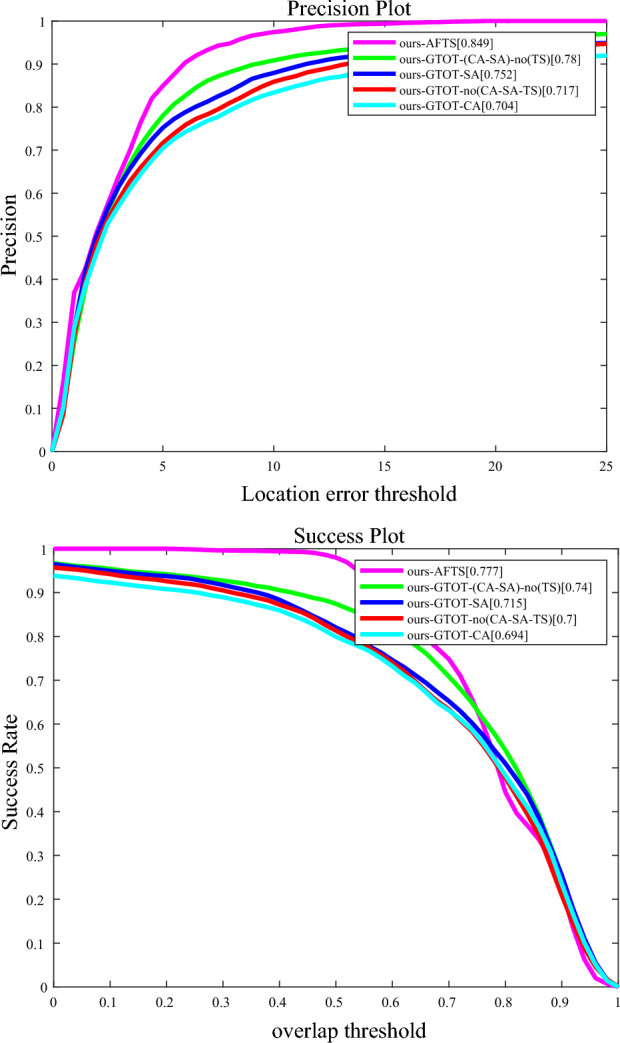
Results of Ablation experiment. ours-GTOT–SA represents the experimental results with only the SA module adds ours-GTOT–CA represents the experimental results with only the CA module added. ours-GTOT–no (CA–SA–TS) represents the experimental results without adding any module; ours-GTOT–(CA–SA)-no (TS) represents the experimental results with only the SA and CA module added. ours-AFTS represents the model with all modules included.

As shown in Fig. 13, these are the results of our ablation experiment. In this experiment, we used the RGBT234 dataset as the training set and the GTOT dataset as the test set. From the graph, we can obtain the following information: the experimental results without adding any module are significantly worse compared to the experimental results with the modules added. In the PR score graph, the experimental results of "ours-GTOT-SA" and " ours-GTOT-CA" are significantly better than the experimental results of "ours-GTOT-no (CA-SA-TS)", indicating that adding the SA and CA modules helps improve the accuracy of the tracker. In the SR score graph, we found that the experimental results of "ours-GTOT-SA" are better than those of " ours-GTOT-no (CA-SA-TS)". However, the experimental results of "our-ca" have decreased compared to "ours-GTOT-no (CA-SA-TS)", indicating that adding the CA module does not help improve the success rate of the tracker, but it still has an effect on improving the accuracy of the tracker. In this experiment, the experimental results of "ours-AFTS" are the highest, indicating that the TS module has a significant impact on improving the success rate of the tracker.

## Conclusion

This paper introduces a novel approach for RGBT tracking, specifically an adaptive tracker based on the Transformer model with dual-Siamese architecture and anchor-free design.

The proposed method incorporates Transformer attention mechanism to replace the correlation operation in the Siamese network, leading to improved tracking success rate. By eliminating candidate boxes and reducing human-induced interference, our approach effectively addresses the limitations of Anchor-Based methods while eliminating the need for many hyperparameters. Experimental results demonstrate the reliability of the proposed algorithm, which successfully exploits the complementary information from visible light and thermal infrared modalities. As part of our future work, we are exploring the integration of RGBD tracking design, aiming to expand the application scope and enhance the performance in challenging scenarios.

## Data Availability

The datasets used and/or analysed during the current study are available from the corresponding author on reasonable request.

## Abbreviations

Siam AFTS: we propose that we build a new tracker for RGB-T target tracking based on the siamese network, called Anchor-Free based Transformer network System (Siam AFTS)
RGB-T: RGB-T images refer to the fact that each set of images consists of a combination of images of both visible and thermal infrared light modes
